# A 43-Year Follow-Up of Unilateral Harrington Rod Instrumentation and Limited Fusion for Adolescent Idiopathic Scoliosis

**DOI:** 10.7759/cureus.14299

**Published:** 2021-04-05

**Authors:** Jordan Vokes, Emmanuel Menga, Addisu Mesfin

**Affiliations:** 1 Orthopaedics and Rehabilitation, University of Rochester Medical Center, Rochester, USA; 2 Orthopaedics Spine Surgery, University of Rochester, Rochester, USA

**Keywords:** scoliosis, ultra-long follow up, revision spinal fusion, harrington rod, idiopathic scoliosis, lumbar obliquity

## Abstract

Limited unilateral instrumentation has been used in the past in the treatment of adolescent idiopathic scoliosis; however, to our knowledge, there are no reported cases with ultra-long follow up regarding this. Our objective is to report on the 43-year follow-up of limited Harrington rod instrumentation for the treatment of a double major adolescent idiopathic scoliosis curve. We describe the patient’s initial presentation, including history, physical exam, radiographic findings and clinical decision-making. Initial coronal cobb angle measurements before surgery were: 14° T1-T5, 42° T5-T12, 44° T12-L4. At 43 years of follow up, there was progression (14°>24°, 42°>70°, 44°>50°) of the patient’s double major scoliosis curve despite unilateral, limited Harrington rod instrumentation from L4-S1. The patient was treated with a T3-pelvis instrumentation and fusion and posterior column osteotomies. To our knowledge, this is the longest follow-up and subsequent revision of a patient undergoing limited, unilateral Harrington rod instrumented fusion for the treatment of a double major adolescent idiopathic scoliosis curve.

## Introduction

Double major idiopathic scoliosis with an oblique take-off of the L5 vertebrae is usually managed without fusion to the sacrum in adolescent patients. Previous techniques have utilized the Harrington distraction rod that was developed in 1962 [1] and did demonstrate some success [2]. Modern-day instrumentation techniques including pedicle screws with three-column fixation may allow for selective thoracic fusion with possible fusion to the mid-lumbar spine for patients with adolescent idiopathic scoliosis (AIS). In this case report, we present an ultra-long follow up of a patient with double major idiopathic scoliosis initially managed with limited L4-S1 fusion and Harrington rod distraction at the convexity of the L4-S1 curvature.

## Case presentation

A 55-year-old female presented to our clinic with low back pain and bilateral leg pain, right greater than left. Her history was significant for a diagnosis of scoliosis at our institution 45 years ago (1974). Her Cobb angle measurements at presentation in 1974 were: left T1-5 14º, right T5-12 42º, left T12-L4 44º, and right L4-S1 18º. Considering the patient’s age of 12 years at the time of initial presentation and risk of continued curve progression, bracing was recommended and the patient was treated with a Milwaukee brace for 23 hours per day for approximately eight weeks. Radiographs at follow up with bracing demonstrated minimal curve correction in the brace. Bending radiographs were obtained and demonstrated structural wedging and no opening of the L5-S1 disc space.

The patient was admitted to the hospital and underwent serial casting. Approximately four months into her casting, the patient was transitioned to a turnbuckle plaster cast at the level of L4 to sacrum followed by a limited surgery including a unilateral left sided L4-sacrum posterior spinal fusion using Harrington distraction rods, hooks and iliac crest bone graft. After surgery, casting was continued for at least four months, at which point no further documentation is available. At four-month post surgery, the patient had radiographs demonstrating a T5-T12 curve of 26º and a T12-L4 curve of 22º.

**Figure 1:**
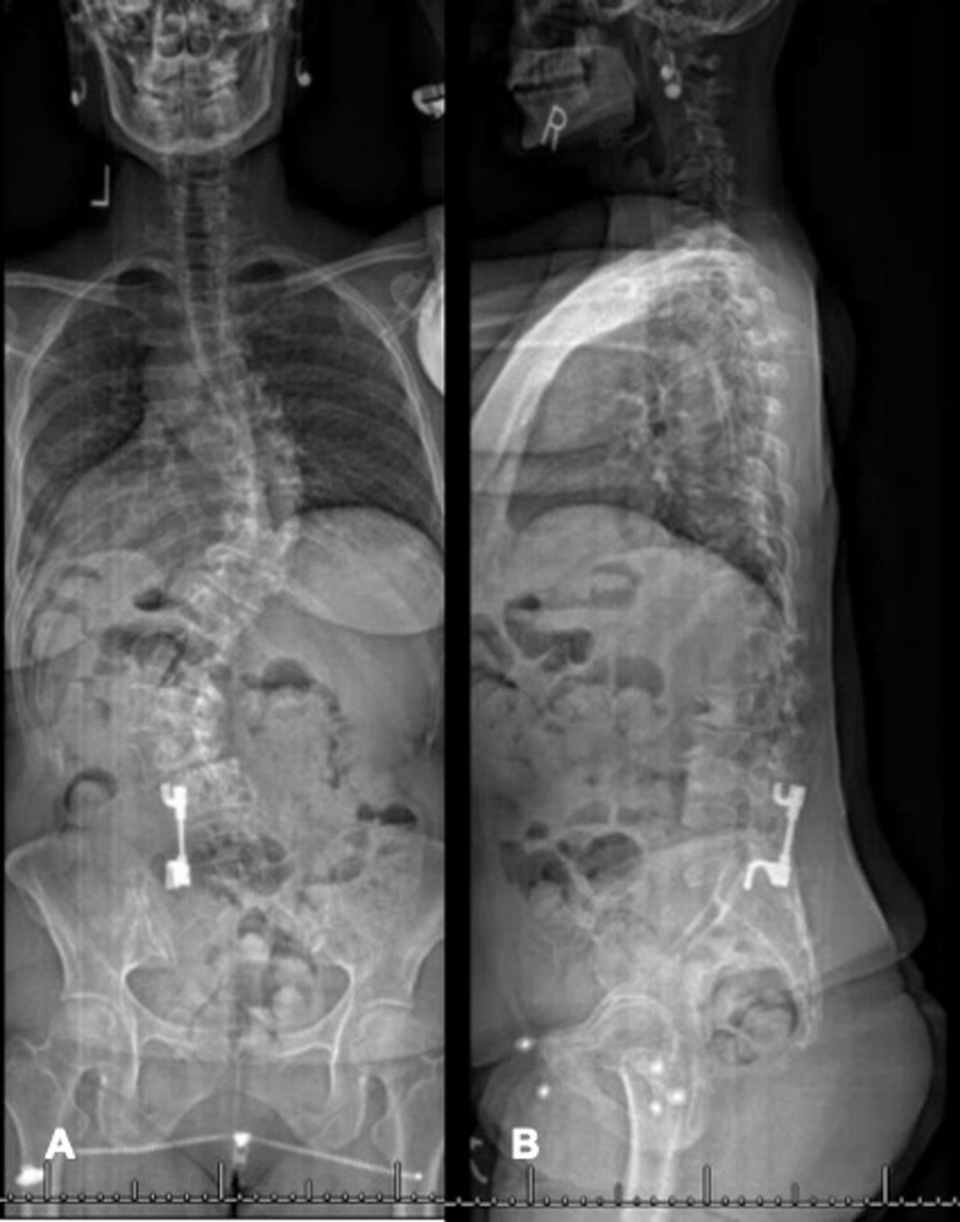
(A) AP and (B) lateral scoliosis X-ray of unilateral L4-sacrum instrumentation for treatment of adolescent idiopathic scoliosis. AP- anteroposterior

Upon re-presentation to our clinic 43 years later, the patient’s scoliosis films demonstrated intact left-sided posterior spinal instrumentation from L4 to the sacrum (Figure 1).

**Figure 2:**
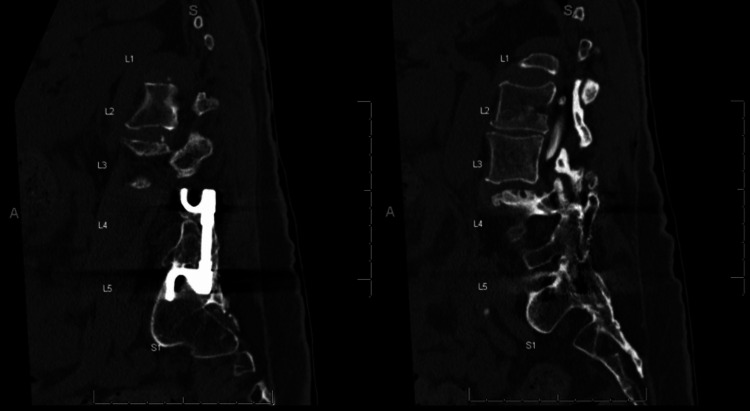
Sagittal CT scan demonstrating Harrington rod instrumentation with intact fusion mass. CT - Computed tomography

The following Cobb angles were measured: left T1-T5 24°, right T5-T12 70°, and left T12-L4 50°, demonstrating further curve progression since her posterior spinal fusion procedure. Her sagittal parameters at that time were as follows: thoracic kyphosis: 8°, lumbar lordosis: 14°, T1 pelvic angle (T1PA): 25°, pelvic incidence (PI): 55°, sagittal vertical axis (SVA): 2.5 cm, PI - lumbar lordosis (PI-LL) = 39°. The patient reported gradually worsening back pain with radiculopathy that was worse on the right. She also expressed cosmetic concerns of sagging on the left side of her back, as well as challenges working due to constantly needing to change jobs due to pain. At this presentation, she had been chronically taking narcotic pain medications and was taking medication for depression. Her magnetic resonance image (MRI) demonstrated severe central canal stenosis with marked right foraminal narrowing at L3-4 along with right neuroforaminal narrowing at L1-2 and L2-3 consistent with her clinical diagnosis. The left neural foramen and central canal imaging was partially obscured by metal artifact. Initial treatment including physical therapy, oral analgesics and selective nerve root injections provided minimal improvement of her symptoms. Computed tomography (CT) demonstrated an intact fusion mass (Figure 2).

**Figure 3:**
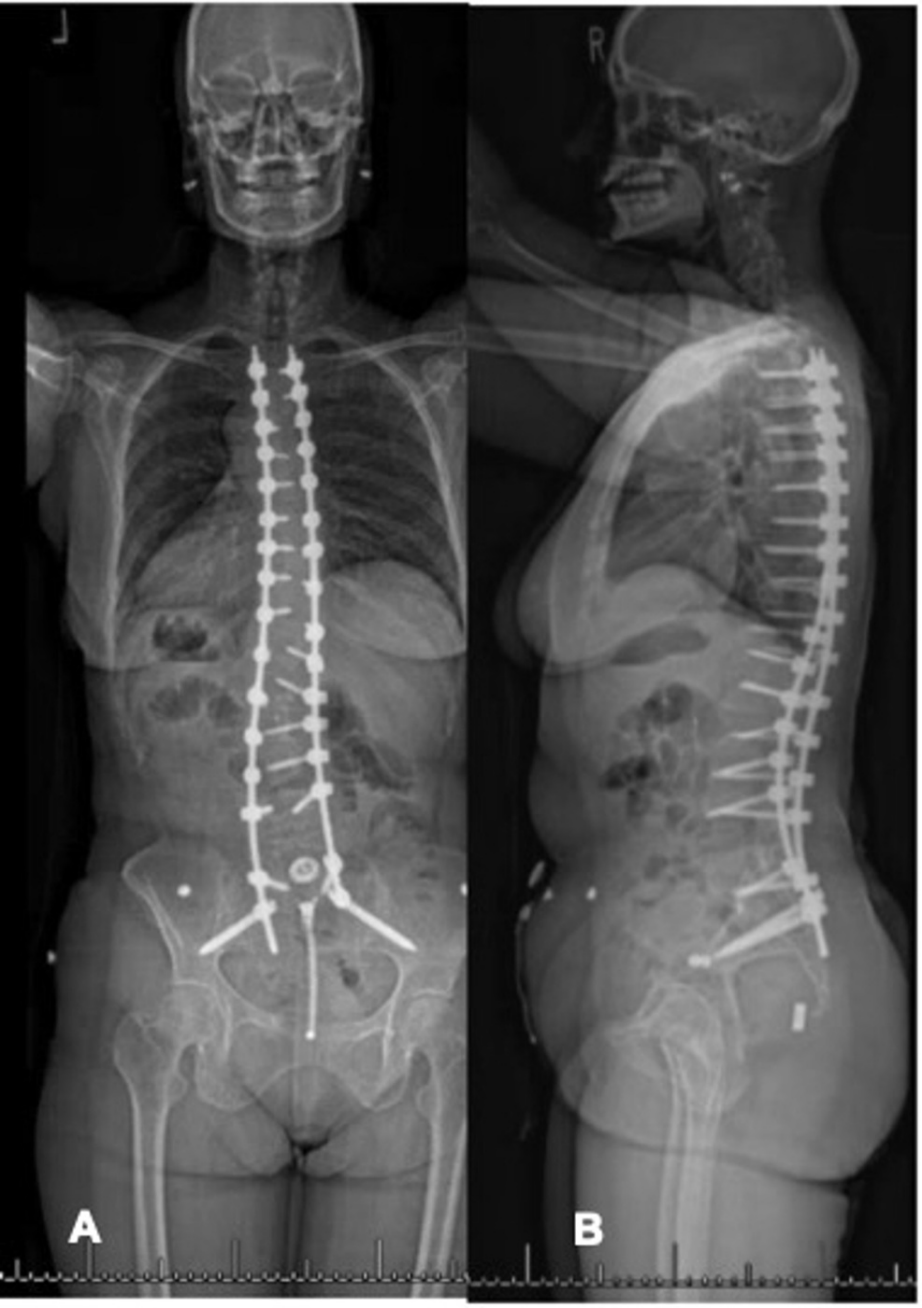
(A) AP and (B) lateral scoliosis X-ray status post T3-pelvis instrumentation and fusion. Post-operative sagittal parameters: thoracic kyphosis: 25°, lumbar lordosis: 31°, T1 pelvic angle (T1PA): 16°, pelvic incidence (PI): 50°, sagittal vertical axis: 0 cm, PI - lumbar lordosis (PI-LL) = 19°.

Due to failure of non-operative management, surgical options were discussed, and the patient underwent a T3-pelvis posterior instrumented spinal fusion with multi-level posterior column osteotomies (T7-10, T12-L4) and laminectomies (L3-S1) (Figure 3). Her post-operative sagittal parameters were: thoracic kyphosis: 25°, lumbar lordosis: 31°, T1PA: 16°, PI: 50°, SVA: 0 cm, PI - lumbar lordosis (PI-LL) = 19°. Her immediate post-operative course was complicated by a cerebellar cerebrovascular accident (CVA) that was felt to be from post-operative hypotension and hypoperfusion. Advanced imaging demonstrated a posterior fossa infarct in the region of the right posterior inferior cerebellar artery (PICA). She was evaluated and managed by the neurology and neurosurgery teams and fortunately made a full recovery with medical management. At the latest follow up, 46 years since her index surgery, the patient was doing well with resolved radicular symptoms. She was able to return to activities such as weight lifting, running, and biking.

## Discussion

This report represents an ultra-long follow up on unilateral, limited posterior instrumented spinal fusion for the treatment of AIS. Based on our results, unilateral instrumented fusion may not be an effective treatment for AIS. Our patient returned for evaluation due to worsening symptoms and progression of her curve despite unilateral instrumented fusion. This was ultimately treated with T3-pelvis (bilateral S2 iliac screws) instrumentation and fusion, with the resolution of her back pain and radicular symptoms and return to a light treadmill and weight-lifting activity at six-month post revision surgery.

The Harrington rod was first developed in 1962 [1]. Segmental spinal instrumentation was developed with the goal of achieving some degree of spinal deformity correction [3]. Surgical goals of scoliosis correction remain the same today as they did many years ago including; halting curve progression, achieving fusion, and protecting and preserving neurologic function. Without adequate treatment, scoliosis can have several appearance related effects including a rib hump, waist asymmetry, shoulder imbalance, and even cardiopulmonary compromise in severe cases [4]. The first attempts at halting curve progression were carried out by Russell Hibbs (1911) who attempted in-situ fusion for Pott’s disease. However, this technique was associated with high non-union rates, high infection rates and little to no correction, as well as prolonged casting and bed rest. Harrington, who first attempted instrumented fusion using concave rod distraction followed by convex rod compression, carried out the next attempt. Unfortunately, Harrington rods demonstrated limited stability, which necessitated long periods of postoperative immobilization [4]. Later came the development of the pedicle screw, first described by Roy-Camille. This technique provided three-column fixation and allowed for single vertebral body manipulation that did not require the laminae and did not require distraction for stability [3]. Suk et al. subsequently demonstrated their effectiveness in the treatment of scoliosis, and pedicle screws have now become the workhorse in the treatment of AIS, today remaining the standard of care in the surgical treatment of AIS [5,6].

Long-term follow-up studies after Harrington instrumentation for the treatment of AIS have overall demonstrated favorable results, with quality of life outcomes similar to the normal population [2,7]. However, there is some evidence to suggest that there is a significant difference in the psychological quality of life, which may be secondary to insufficient correction of the patient’s cosmetic deformity with Harrington instrumentation [7]. More recent literature has demonstrated that pedicle screw instrumentation is more effective and provides greater curve correction than segmental hook instrumentation in the treatment of AIS [8-10]. There is literature to support the use of unilateral instrumentation to achieve spinal fusion in both degenerative conditions of the lumbar spine and spinal deformity [11-15]. In a recent systematic review, the majority of published reports regarding unilateral instrumentation for degenerative spine conditions involved only a single lumbar level [11]. The use of unilateral pedicle screw instrumentation for spine deformity has been described by Tsirikos et al. who reported a single-center series of multi-level unilateral instrumentation (pedicle screws/hook-rod construct) for scoliosis with minimal complications in selected patients [12]. The same group has also reported the use of low-density pedicle screws on the concavity of the curve (proximal/distal aspect of the construct) and instrumenting most levels on the convexity of the curve [13,14]. Either of these techniques may be sufficient when addressing thoracic or thoracolumbar curves; however, scoliosis with L5 obliquity would be challenging to address with unilateral instrumentation as demonstrated in our long-term follow-up.

The clinical effects and manifestations of scoliosis can be significant. In our case, the patient returned 43 years after her index operation with persistent cosmetic concerns of sagging on the left side of her back, and she reported challenges with her job leading her to change jobs secondary to pain. Her history was also significant for a diagnosis of chronic narcotic use in order to control her pain and depression that was managed with citalopram. It is difficult to ascertain whether a more traditional initial operation would have resulted in a better long-term outcome, but there is evidence to support favorable outcomes after spinal fusion with a single Harrington distraction rod at 20-year follow up [15].

Limitations

There are limitations to our study. After the patient’s initial surgery and subsequent casting, she was lost to follow up for over 40 years. It is not entirely clear whether the patient had sought any follow up for which we do not have documentation or if any further surgical intervention was recommended prior to her return to our clinic. In addition, this is the only known case of ultra-long follow-up, to our knowledge, of a limited, unilateral fusion for the treatment of AIS and it is unknown whether other patients were treated in a similar fashion with similar or different outcomes.

## Conclusions

In summary, to our knowledge, this is the longest follow up of a patient undergoing limited, unilateral Harrington rod instrumented fusion for the treatment of a double major AIS curve. Based on our results, this technique is inadequate and ineffective for the treatment of this condition as it fails to adequately limit further curve progression.
